# Differential Impact of Spike Protein Mutations on SARS-CoV-2 Infectivity and Immune Evasion: Insights from Delta and Kappa Variants

**DOI:** 10.4014/jmb.2411.11001

**Published:** 2024-12-02

**Authors:** Tae-Hun Kim, Sojung Bae, Jinjong Myoung

**Affiliations:** Korea Zoonosis Research Institute, Department of Bioactive Material Science and Genetic Engineering Research Institute, Jeonbuk National University, Jeonju 54531, Republic of Korea

**Keywords:** SARS-CoV-2, infectivity, immune evasion

## Abstract

SARS-CoV-2 continues to pose a global health challenge due to its high transmissibility and mutability, with new variants emerging that potentially undermine vaccination and therapeutic efforts. Mutations in the spike protein, particularly in the receptor-binding domain (RBD), significantly influence viral transmissibility and immune escape. However, the complex interplay of these mutations and their combined effects on viral fitness remain to be analyzed. In this study, we investigated the functional impact of key mutations found in the Delta and Kappa variants of SARS-CoV-2. Using pseudovirus assays, we demonstrated that the T478K and L452R mutations characteristic of the Delta variant primarily enhance viral infectivity, with minimal effect on antibody-mediated neutralization. Conversely, the E484Q mutation of the Kappa variant, alone or in combination with L452R, significantly improved evasion of antibody-mediated neutralization but appeared to compromise viral fitness and infectivity. Notably, contrary to previous reports, we found that the P681R mutation contributed neither to increased infectivity nor immune evasion at least in the assay system employed in this study. Our findings suggest that the Delta variant's global dominance over the Kappa variant may be attributed to its superior infectivity and transmissibility rather than enhanced immune evasion capabilities. These results provide valuable insights into the functional consequences of spike protein mutations and may aid in predicting the emergence and spread of future SARS-CoV-2 variants. Such understanding is crucial for enhancing public health preparedness and informing the development of next-generation vaccines and therapeutics.

## Introduction

Since its initial appearance in 2019, SARS-CoV-2, the causative agent of COVID-19, has caused over 700 million infections and 7 million deaths globally [1]. SARS-CoV-2 belongs to betacoronavirus and is classified as a positive-sense RNA virus with a single strand and an approximate genome size of 30,000 bases [2]. Due to the fact that it encodes exonuclease N (non-structural protein 14, nsp14), which performs the function of proof-reading [3, 4], SARS-CoV-2 is hypothesized to undergo less radical evolution than other RNA viruses [5]. Nevertheless, due to the fact that it infects wild and domesticated animals (minks, white-tailed deer, etc.) [6-8] and hundreds of millions of people during pandemics, a considerable number of variants have been identified and temporarily established dominance in human populations before being replaced by another variant within months of its emergence [9, 10].

The SARS-CoV-2 delta variant was first detected in October 2020 and quickly replaced the previous dominant strain, the Kappa variant in India, eventually spreading to the rest of the world [11, 12]. It has been shown to cause diseases with higher severity and to be associated with an increased risk of death [13]. In a large-scale study conducted by a Canadian research team [14], analyzing more than 200,000 COVID-19 cases, showed that the Delta variant may cause 108% increased risk for hospitalization, 235% increased risk for intensive care unit (ICU) and 133% increased risk for death compared to the Wuhan strain. It is significantly higher than other variants: the beta and gamma, 51% more likely to die of the infection than those who caught the Wuhan.

An initial apprehension surrounding the evolution of SARS-CoV-2 was the possible appearance of antigenically unique variants capable of eluding immunity acquired through vaccination or infection, as demonstrated by the N439K spike substitution [15]. Many of prominent COVID-19 vaccines, with just a few mutations in the spike to take up a prefusion conformation [16], relied on the spike antigen of Wuhan-Hu-1. In laboratory experiments, Beta, Gamma, and Delta exhibited a moderate degree of evasion from vaccine-derived antibodies and convalescent sera [17, 18], whereas Alpha exhibited minimal antigenic change [19, 20]. Therefore, first-generation SARS-CoV-2 vaccines, despite being developed using an extremely early spike sequence, seemed to effectively protect against severe disease [21-25]. Thus, it is conceivable that determining factor(s) for establishing predominance by a variant replacing the previous ones may involve higher infectivity rather than antibody escape [26, 27]. In fact, many previous papers described that the Delta variant displayed higher infectivity and pathogenicity than previous dominant variants, namely Alpha, Beta, and Gamma variants. However, its underlying molecular mechanisms remain unclear. In addition, determination of the roles of mutations found in the receptor binding domain (RBD) of the spike of the Delta variant need further scrutiny. To determine relative importance of infectivity vs immune escape in the determination of the dominance between the Kappa and the Delta variant, and to further analyze the roles of mutations in the dominancy transition, singly or in combinations, we generated a series of pseudoviruses bearing mutations found in the RBD of the Kappa and Delta spike. In the case of the Kappa variant, E484Q mutation was found to be responsible for enhanced immune escape but not for infectivity. On the other hand, T478K and L452R mutations, only in combination, significantly enhanced viral infectivity of the Delta variant with minimal effect, if any, on antibody escape with therapeutic antibodies tested in our study. Taken together, our data suggest that viral infectivity, rather than immune escape, may provide a molecular explanation for the changes of dominance from the Kappa to the Delta variant.

## Materials and Methods

### Cells and Reagents

HEK 293T cells (ATCC, CRL-3216) were grown in Dulbeccós Modified Eagle Medium High Glucose (DMEM; Welgene, Republic of Korea) supplemented with 10% Fetal Bovine Serum (FBS; Welgene, Republic of Korea) and 1% Penicillin-Streptomycin (P/S; Welgene). HCC15-ACE2 cells, which are lentiviral transduced HCC15 cells (a lung carcinoma cell line; ATCC, CCL-225), are a stable cell line which expresses cellular receptor of SARS-CoV-2. The stable cells were grown in media supplemented with 10% FBS and 1% P/S from Roswell Park Memorial Institute 1640 (RPMI 1640; Welgene). Vero E6 cells (ATCC, CRL-1586) were grown in DMEM supplemented with 10% FBS and 1% P/S. HEK 293T, HCC15-ACE2 and Vero E6 cells were maintained at 37°C and 5% CO_2_.

### Visualization of SARS-CoV-2 Spike Protein and Mutations Using PyMOL

The SARS-CoV-2 spike structure was obtained from the RCSB Protein Data Bank (PDB) under accession number 6VXX. This structure was resolved at 2.8 Å resolution using cryo-electron microscopy (cryo-EM). The 6VXX-based spike protein structure was visualized and its structural features and introduced mutations were highlighted using PyMOL molecular visualization software. The spike protein structure was depicted in cartoon format to clearly represent its secondary structural elements such as helices and sheets. For clarity, the spike protein's key functional regions were color-coded: the N-terminal domain (NTD, residues 13–304) was shown in green, the receptor-binding domain (RBD, residues 319–541) in red, the receptor-binding motif (RBM, residues 438–508) in yellow, the fusion peptide (residues 788-806) in purple, and the heptad repeat regions (HR1 and HR2, residues 918–983 and 1162–1203, respectively) in light beige. Other regions of the spike protein were colored gray to distinguish them from the highlighted domains. Mutations introduced into the spike protein were represented as distinct dot annotations on the structure.

### Mutagenesis of the Spike Protein of the SARS-CoV-2 Wuhan Strain

The spike gene of SARS-CoV-2 was optimized for mammalian expression by the GeneArt (Thermo Fisher Scientific, USA), and cloned into pcDNA3.1 (Invitrogen). To generate Spike proteins with one or more mutations, inverse sequence and ligation independent cloning (SLIC) was performed [28-31] with the primers listed below, with mutated codons highlighted. K417N forward; 5'-CAGGCAACATCGCCGATTACAACTACAAGCTGCCCGAC-3', K417N reverse; 5'-CGGCGATGTTGCCTGTCTGTCCAGGAGCGATCTGC-3', E484K forward; 5'-GGC GTGAAGGGCTTCAACTGCTACTTCCCACTGCAG-3', E484K reverse; 5'-GAAGCCCTTCACGCCGTT ACAAGGGGTGCTGCCG-3', N501Y forward; 5'-CCTACCTACGGCGTGGGCTATCAGCCCTATAGAGTGGTG-3', N501Y reverse; 5'-CACGCCGTAGGTAGGCTGAAAGCCGTAGGACTGCAG-3', D614G forward; 5'-TATCAGGGCGTGAACTGTACAGAGGTGCCCGTGG-3', D614G reverse; 5'-GTTCACGCCCTGATACAG CACGGCCACCTGATTG-3'. The expression of the spike protein, harboring indicated mutations, was analyzed by Western blotting using polyclonal anti-spike S2 antibodies (SinoBiological, China).

### Generation of Lentivirus Pseudo-Typed with SARS-CoV-2 Spike Protein

Pseudo-typed lentiviruses, expressing the SARS-CoV-2 spike protein with or without mutations found in the spike protein of Kappa and Delta variants, were generated by co-transfecting HEK293T cells with plasmids expressing HIV1 gag-pol (psPAX2), SARS-CoV-2 spike (with or without mutations), a lentiviral vector backbone (pHR encoding firefly luciferase) as described before [32, 33]. The Trans*IT*-Lenti Transfection Reagent (Mirus Bio LLC, USA) was used for DNA transfection. At 72 h post-transfection, culture supernatants containing pseudoviruses were harvested with subsequent removal of debris by centrifugation (3,600 RPM for 5min at 4°C). Pseudoviruses were kept at -80°C until use. Pseudoviruses were titrated using quantitative RT-PCR (qRT-PCR). Briefly, pseudoviral RNA was extracted and purified using the QIAamp Viral RNA Mini Kit (Qiagen, Germany), and subsequently subjected to single-step qRT-PCR using the Lenti-X qRT-PCR Titration Kit (Takara, Japan) according to the manufacturer's instructions [34].

### Measurement of Pseudovirus Infectivity

Pseudoviruses were titrated and normalized to ensure that the same numbers of virus particles were used for infectivity test before infected into HCC15-hACE2 cells, which are a lung carcinoma cell line stably expressing hACE2, supports high levels of SARS-CoV-2 and pseudoviral infection, making it suitable for analyzing the viral entry process [35]. Pseudovirus-infected cells were lysed at 72 h post-infection for 5 min on ice with chilled Reporter Lysis 5X Buffer (Promega, USA), and then precipitated by centrifugation (at 15,000 RPM for 15 min at 4°C). 25 ul of each lysate was transferred to a new tube, and mixed with 25 ul of Luciferase buffer containing luciferase substrate (Luciferase Assay System, Promega) [36, 37].

### SARS-CoV-2 Variant Viruses

SARS-CoV-2 kappa (B.1.617.1) and delta (B.1.617.2) variant viruses were obtained from the Korea Center for Disease Control (KCDC #43389 and #43390, respectively) and amplified once in Vero-E6 cells before use at MOI 0.1. Viruses in the culture supernatants were harvested at 60 hr post-infection and virus stocks were titrated by tissue culture infectivity dose 50 (TCID50) as previously described [38, 39].

### Neutralization Assay

Either pseudoviruses or live SARS-CoV-2 delta or kappa variant viruses were mixed with indicated monoclonal antibodies alone or in combination at 37°C for 1 h before infection into Vero E6 cells. Cells were then extensively washed and incubated for 6 h (live SARS-CoV-2 virus) or 72 h (Pseudoviruses) before being subjected to quantitative RT-PCR or luciferase assay, respectively. The percentage of neutralization was calculated using the following formula: (value(untreated)-value(treated)) × 100/value(untreated) (%). Data were plotted using Origin 8.5, and polynomial regression analysis was performed to calculate the inhibitory concentration 50 (IC_50_).

### Quantitative Real-Time Reverse Transcription Polymerase Chain Reaction (qRT-PCR)

The RNA was extracted using the GENTI advanced Viral RNA Extract Kit and the GENTI 32 Advanced Automatic Extraction Equipment (GeneAll, Republic of Korea) [40-43]. 140 μl of extracted RNA was used in a qRT-PCR assay with the DiaStar OneStep Multiplex qRT-PCR kit (SolGent, South Korea) with the following set of primers and probe employed: forward primer; 5’-GACCCCAAAATCAGCGAAAT-3’, reverse primer; 5’-TCTGGTTACTGCCAGTTGAA-3’, probe; 5’(FAM)-CGCATTACGTTTGGTGGACCCTCA-(TAMRA)3’.

### Statistical Analysis

Statistical analysis was performed using Student’s *t*-test. Statistical significance was defined as a probability value (*p*-value) of lower than 0.05 (*, *p* < 0.05; **, *p* < 0.01; ***, *p* < 0.001; ns, *p* ≥ 0.05). Data are presented as the mean ± standard deviation (SD).

## Results

### The Presence of Both T484K and L452R Mutations in the Spike of the Delta Variant Significantly Enhances Infectivity of Pseudo-Typed Viruses

**Fig. 1:**
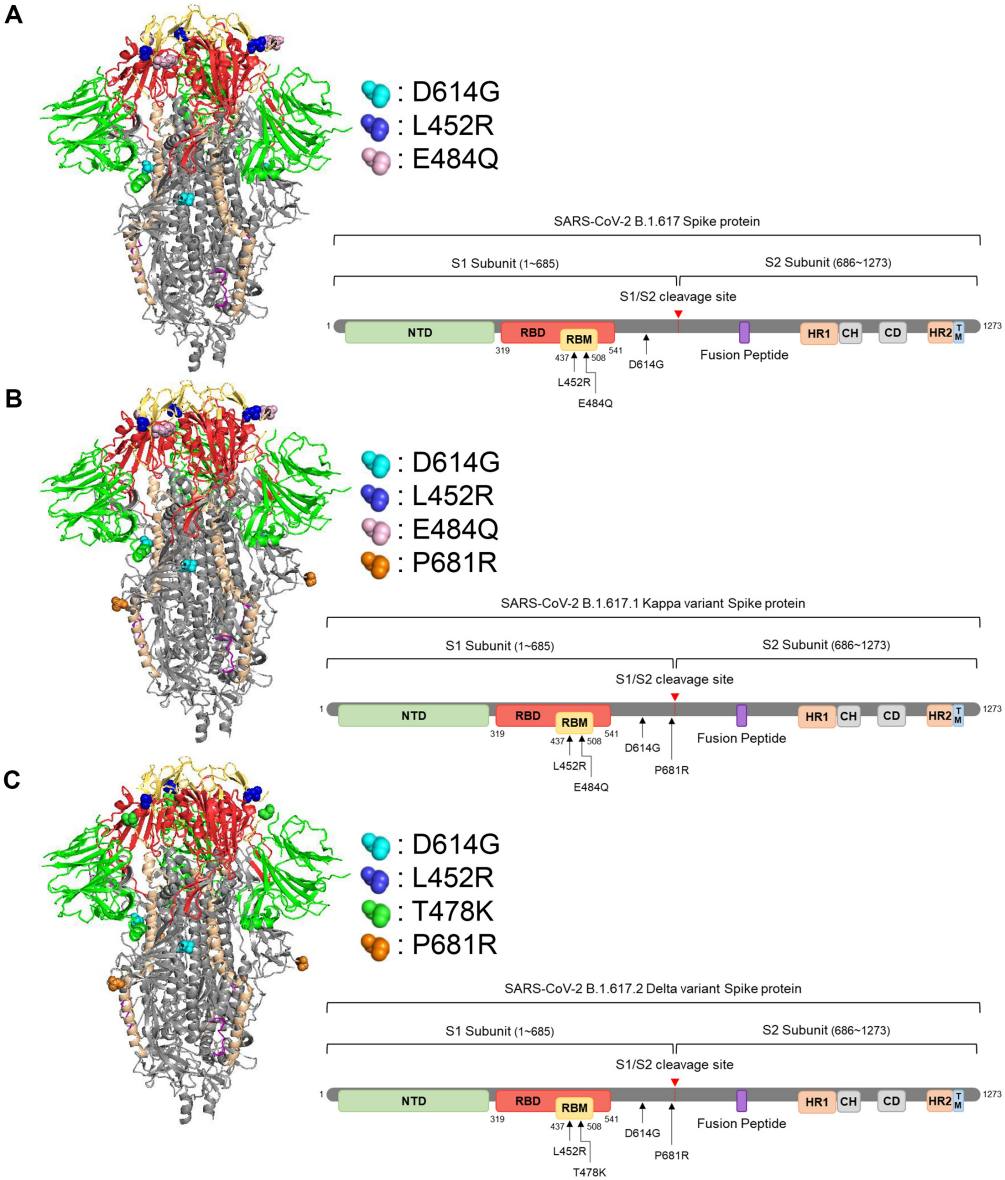
Structural comparison of kappa and delta variant of SARS-CoV-2. The structural coordinates of the Wuhan-Hu-1 reference spike protein were obtained from the RCSB Protein Data Bank (PDB ID: 6VXX). Predicted structures of the Kappa (B.1.617.1) and Delta (B.1.617.2) variant spike proteins were generated using PyMOL (Schrödinger, LLC). Key mutations in the variant spike proteins are highlighted and labeled. The receptor-binding domain (RBD) is shown in red, while the N-terminal domain (NTD) is depicted in green. Mutations are represented as spheres and colored according to their domain location.

**Fig. 2:**
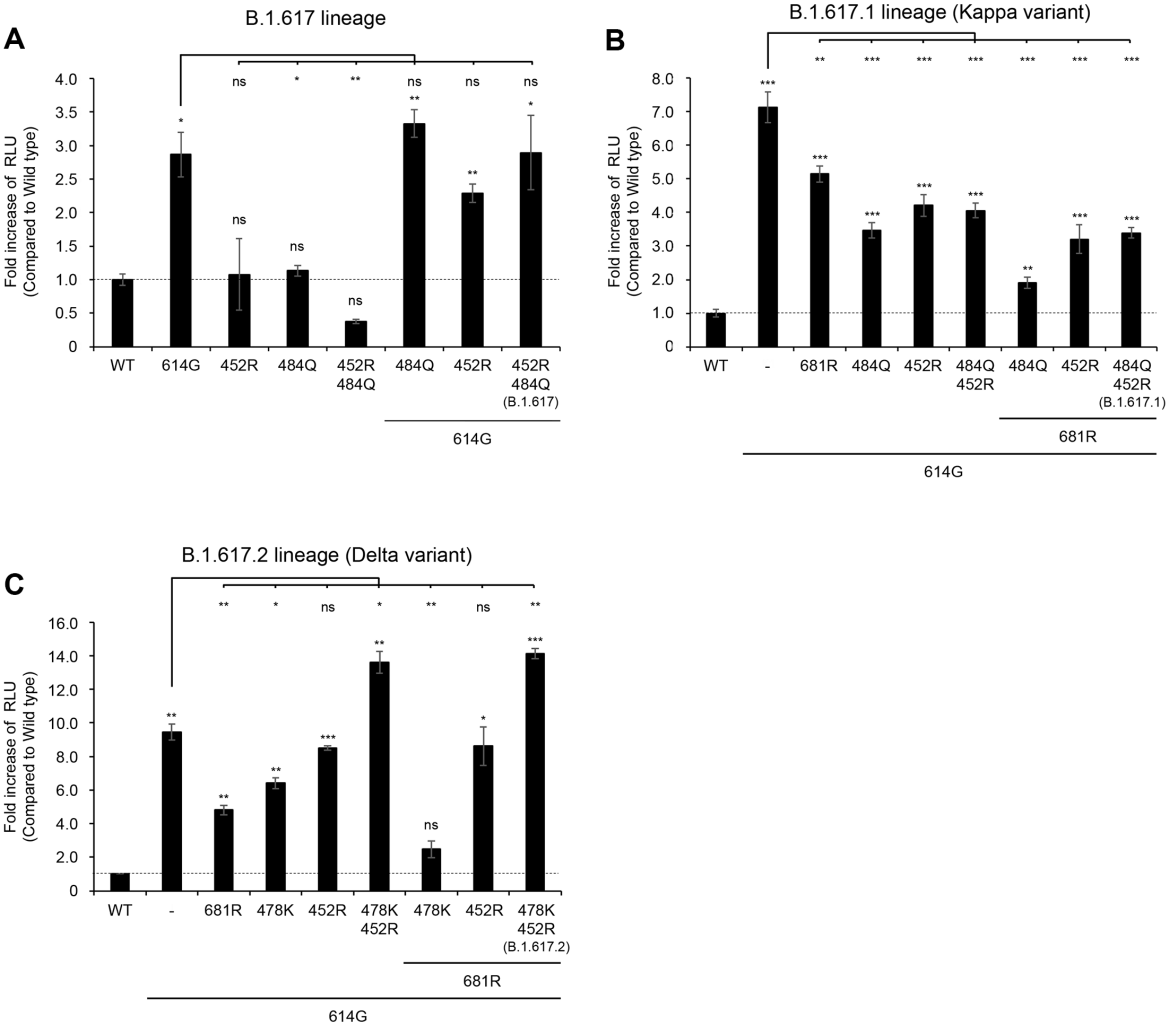
Synergistic effect of T478K and L452R mutations on SARS-CoV-2 Delta variant pseudovirus infectivity. ACE2-expressing target cells were infected with pseudoviruses bearing wild-type (WT) or variant spike proteins. At 72 h post-infection, cells were lysed and subjected to a luciferase assay. Luciferase activity was normalized to that of the WT spike, and the fold difference in relative luciferase units (RLU) compared to WT is presented. Data shown are representative of three independent experiments. Error bars represent mean ± SEM of technical replicates. Statistical significance was determined using one-way ANOVA with Dunnett's post-hoc test. *p < 0.05, **p < 0.02, ***p < 0.001 compared to WT.

The Kappa and Delta variants emerged roughly at the same time in India by December 2020[44]. The Kappa variant prevailed first, accounting for more than half of the SARS-CoV-2 infections in the country by March 2021. However, it was replaced later with the Delta variant which rapidly spread to over 179 countries by November 2021. The spike of the Delta variant demonstrated higher affinity to the viral receptor (ACE2), and both the capability of immune escape and increased ACE2 binding were suggested as explanations for the rapid dominance of the Delta variant [44]. As the spike of the Kappa and the Delta variant encodes common and unique mutations in their receptor-binding domain (RBD), we set out to determine which mutation(s) in the spike of the Delta variant contribute to increased infectivity of Delta, thus taking-up of global prevalence. We generated a series of pseudo-typed viruses encoding a single or combinations of each mutation, which are found in the RBD L452R (B.1.617, Kappa, and Delta), E484Q (B.1.617 and Kappa), T478K (Delta) or its vicinity (D614G and P681R). For details, see Fig. 1 and Table 1. D614G mutation, when introduced in the Spike, significantly enhanced pseudo-typed viruses in the case of B.1.617, B.1.617.1 (Kappa), and B.1.617.2 (Delta), which is consistent with previous data [35, 45-47]. However, interestingly, the presence of a single or combinations of L452R, E484Q, and P681R mutation (B.1.617 and Kappa) did not further enhanced viral infectivity. But rather, most of cases, introduction of mutations, singly or in combination, reduced the infectivity of pseudo-typed viruses, suggesting that viral fitness was not optimal (Fig. 2A and 2B). In contrast, in the case of Delta mutations, introduction of both T478K and L452R mutations further enhanced D614G-mediated increase of infectivity of pseudo-typed viruses while each mutation alone or the presence/absence of P681R mutation had little, if any, effect on viral infectivity (Fig. 2C)

### Both E484Q and L452R Mutations Are Required for Resistance to Neutralization by RBD-Binding Therapeutic Antibodies

**Fig. 3:**
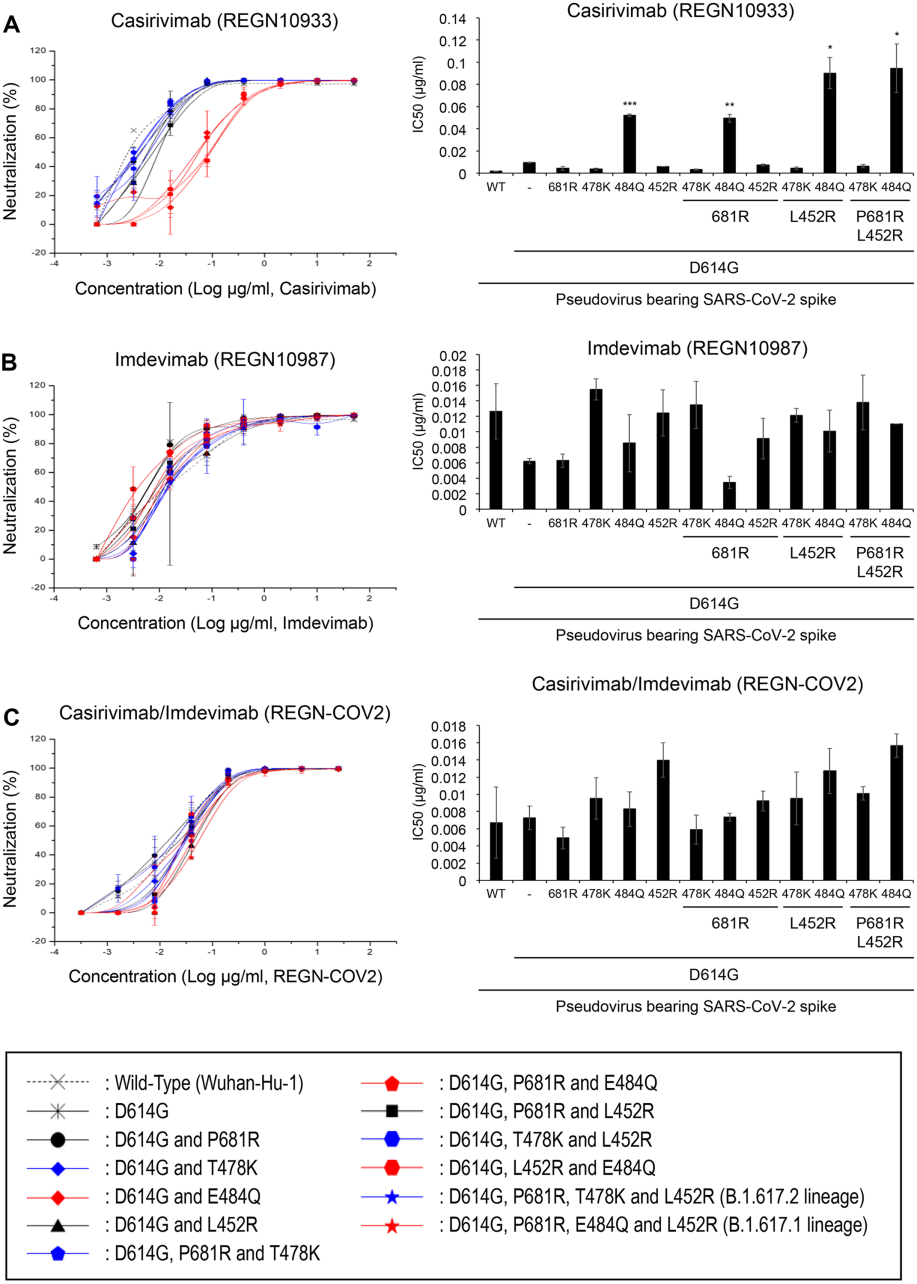
Differential neutralization sensitivity of pseudoviruses encoding mutations of Kappa and Delta variants to Casirivimab (REGN10933). Neutralization assays were performed using pseudoviruses bearing various spike protein mutations. Pseudoviruses were incubated with (**A**) Casirivimab alone, (**B**) Imdevimab alone, or (**C**) a combination of both antibodies at 37°C for 1 h prior to infection of ACE2-expressing target cells. After 72 h, cells were lysed and luciferase activity was measured. Left panels: Dose-response neutralization curves. Data points represent the mean ± SEM of percent neutralization relative to untreated controls from two independent experiments, each performed in triplicate. Right panels: Half-maximal inhibitory concentrations (IC_50_) for each antibody or combination against different pseudovirus variants. Bars represent the mean IC_50_ values from two independent experiments. IC_50_ values were calculated as described in the Materials and Methods section. Figure legends for A, B, and C are shown at the bottom. Statistical significance was determined using [statistical test, *e.g.*, one-way ANOVA with Dunnett's post-hoc test] comparing each variant to the wild-type. *p < 0.05, *p < 0.001, ***p < 0.001.

Establishing prevalence by a virus may entail not only higher infectivity but also immune escape [48]. To test whether the Kappa variant, which acquired national prevalence in India before the Delta variant, is better at immune escape compared to the Delta variant, a series of pseudoviruses, that harbor a single or combinations of mutations in the spike, were employed. Pseudoviruses were mixed with the indicated antibodies (Fig. 3) and incubated at 37°C for an hour before added into cells as described in the Materials and Methods. The percentage of neutralization was calculated and plotted (Fig. 3, left panels). The half-maximal inhibitory concentration (IC_50_) was determined and graphically represented (Fig. 3, right panels). Notably, the E484Q mutation emerged as the primary factor contributing to neutralization escape from Casirivimab, an antibody that targets the receptor-binding domain (RBD). This mutation resulted in an approximate 26-fold increase in IC_50_ compared to the wild-type (WT) strain, with IC_50_ values of 0.052 and 0.002, respectively (Fig. 3A). The presence of the E484Q mutation in the Kappa variant, absent in the Delta variant, suggests a potential competitive advantage for Kappa in terms of antibody evasion. This advantage may have contributed to its initial prevalence. Additionally, the L452R mutation emerged as another significant factor in conferring resistance to Casirivimab-mediated neutralization, as evidenced in Fig. 3A (right panel). The L452 mutation alone proved insufficient for significant antibody evasion. However, when combined with the E484Q mutation, a substantial increase in the IC_50_ value was observed, indicating enhanced antibody escape (IC_50_ = 0.006 vs 0.09, representing a 15-fold difference). In contrast, Imdevimab, which targets a region of the spike protein outside the receptor-binding motif (RBM) [49], exhibited relatively consistent IC_50_ values across the tested pseudoviruses, regardless of their mutational profiles (Fig. 3B). This suggests that the efficacy of Imdevimab remained largely unaffected by the various mutations present in these pseudoviruses. Furthermore, when the pseudoviruses were exposed to a combination of both antibodies, the resulting IC_50_ value pattern closely resembled that observed with Imdevimab alone (Fig. 3C).

### The Kappa Variant Virus Efficiently Escapes Casirivimab-Mediated Neutralization

**Fig. 4:**
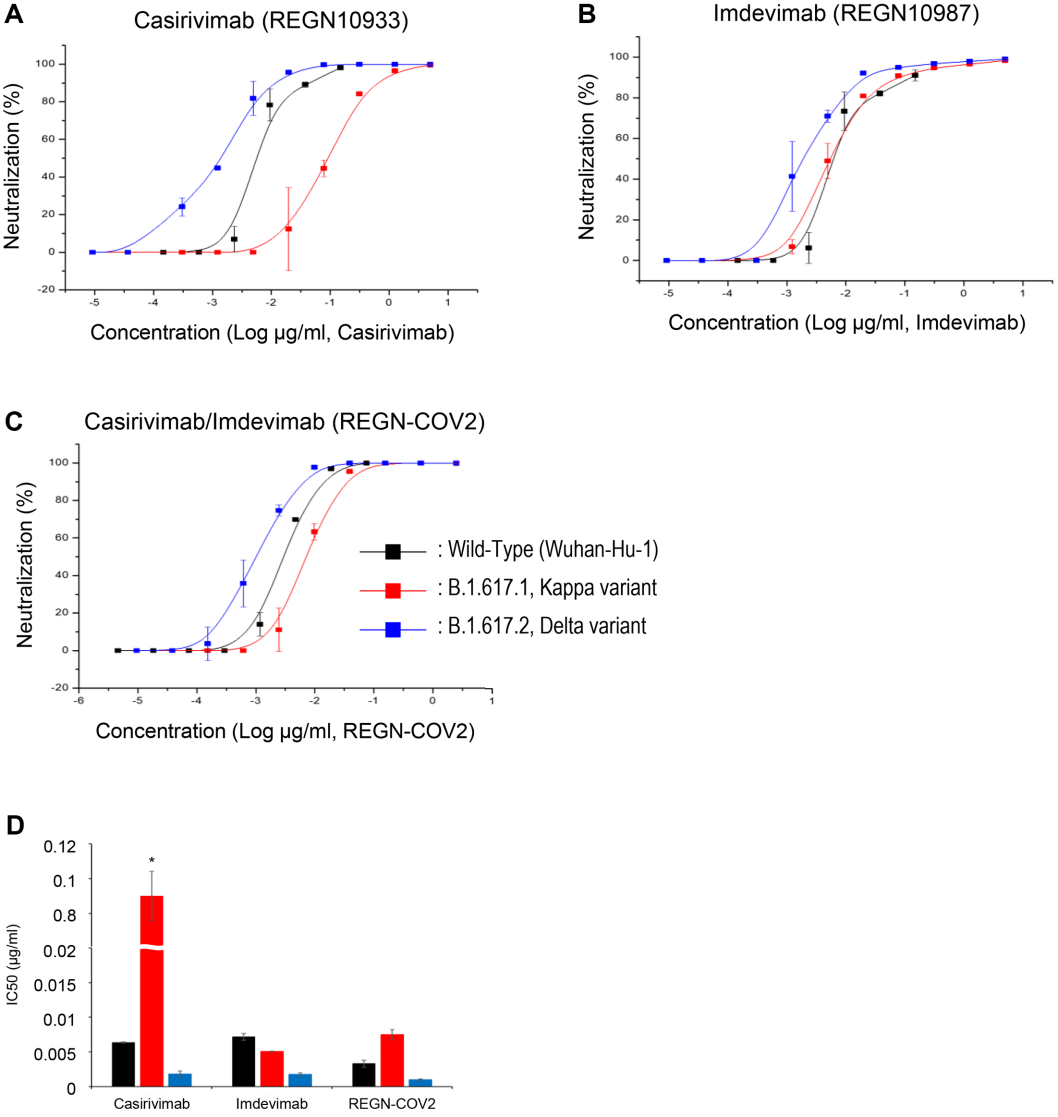
Differential neutralization sensitivity of SARS-CoV-2 variants to Casirivimab (REGN10933). SARSCoV- 2 Wuhan (wild-type), Kappa (B.1.617.1), or Delta (B.1.617.2) variant viruses were incubated with (**A**) Casirivimab alone, (B) Imdevimab alone, or (**C**) a combination of both antibodies at 37°C for 1 hour. After washing, the virus-antibody mixtures were used to infect target cells for 6 h. Intracellular viral RNA was quantified by qRT-PCR and normalized to antibodyuntreated controls. (**A-C**) Dose-response neutralization curves. Data points represent the mean ± SEM of percent neutralization from two independent experiments, each performed in triplicate. (**D**) Half-maximal inhibitory concentrations (IC_50_) for each antibody or combination against different viral variants. Bars represent the mean IC_50_ values from two independent experiments. Statistical significance was determined using one-way ANOVA with Dunnett's post-hoc test comparing each variant to the wild-type. *p < 0.05. Detailed methods for virus preparation, neutralization assays, and qRT-PCR are described in the Materials and Methods section.

Our previous findings demonstrated that pseudoviruses encoding E484Q and L452R mutations exhibited approximately 26-fold greater escape from Casirivimab neutralization compared to those with wild-type sequences. To further investigate this phenomenon, we assessed whether the SARS-CoV-2 Kappa variant would display similar escape efficiency. We incubated Kappa or Delta variant viruses with specified antibodies for one hour at 37°C prior to infecting VeroE6 cells. Intracellular virus titers were quantified via qRT-PCR (methodology detailed in Materials and Methods). Neutralization percentages were calculated (Fig. 4A-4C) and IC_50_ values were extrapolated (Fig. 4D).

Notably, the Kappa variant demonstrated substantial escape from Casirivimab neutralization (IC_50_ = 0.09), whereas Delta variant viruses were effectively neutralized (IC_50_ = 0.0018, Fig. 4A). This suggests that the Kappa variant exhibited approximately 50-fold greater escape from Casirivimab compared to Delta (Fig. 4D). Conversely, both variant viruses showed comparable sensitivity to Imdevimab-mediated neutralization (Fig. 4B). As anticipated, when exposed to both antibodies simultaneously (Fig. 4C), the variants were neutralized as efficiently as with Imdevimab alone. This indicates that the Kappa variant is primarily resistant to Casirivimab, which targets the receptor-binding domain (RBD) of the spike protein.

## Discussion

We employed a pseudovirus system expressing spike proteins with various single or combined mutations in the receptor-binding domain (RBD) region (depicted in Fig. 1). The inherent safety features and experimental flexibility of this system enabled us to conduct investigations in a biosafety level 2 environment [50, 51], allowing for the assessment of each mutation's relative impact on viral transmissibility (Fig. 2) and antibody evasion (Fig. 3). Furthermore, by incorporating multiple mutations within a single spike protein, we were able to analyze their additive or synergistic interactions. In addition, to validate our findings, we compared the data obtained from the pseudovirus system with results from live virus experiments in a biosafety level 3 laboratory (Fig. 4). This comparison demonstrated that the pseudovirus system serves as an effective model for investigating and analyzing the role of individual spike protein mutations in SARS-CoV-2 variants. Consequently, this approach enables us to infer key determinants in the transition of viral dominance from one variant to another. The pseudovirus system thus proves to be a valuable tool in elucidating the complex interplay of mutations that contribute to the evolving landscape of SARS-CoV-2 variants and their impact on viral fitness and immune evasion.

The replacement of the Kappa variant by the Delta variant in India presents a unique opportunity to investigate the determinants of viral dominance [52, 53], particularly the interplay between transmissibility and immune evasion. Our findings, as illustrated in Figs. 2B and 3A, demonstrate that mutations in the receptor-binding domain (RBD) of the Kappa variant reduced pseudovirus infectivity compared to the control (D614G mutation alone). The D614G mutation enhanced pseudoviral infectivity 7-fold relative to the wild-type virus, consistent with previous reports [35, 54, 55]. These data suggest that the mutations investigated in this study, which are encoded by the Kappa variant, do not contribute to increased viral infectivity.

Notably, the E484Q mutation, in conjunction with D614G, significantly reduced viral infectivity compared to D614G alone. This suggests that the dominance of the Kappa variant may not be attributed to enhanced infectivity conferred by E484Q [56, 57], but rather to improved immune evasion. Indeed, our results in Fig. 3 support this hypothesis. E484Q alone was sufficient to evade Casirivimab-mediated neutralization, and this effect was further enhanced in the presence of L452R, indicating a potential synergistic interaction between these mutations in antibody evasion.

Interestingly, L452R alone did not enhance viral infectivity (Fig. 2B and 2C) or antibody evasion (Fig. 3A and 3B) [58, 59], suggesting an auxiliary role for this mutation. These observations lead us to hypothesize that the B.1.617 virus may have initially acquired the E484Q mutation, conferring a competitive advantage through enhanced immune evasion and transmission. This advantage may have subsequently facilitated the acquisition of the L452R mutation, allowing for synergistic interactions between the two mutations. This study provides insights into the evolutionary trajectory of SARS-CoV-2 variants and the complex interplay between mutations that contribute to viral fitness and immune evasion.

In contrast to the Kappa variant, our analysis of the Delta variant revealed that neither T478K nor L452R mutations, individually or in combination, contributed significantly to antibody evasion (Figs. 3 and 4) [53, 60]. However, the co-occurrence of these mutations enhanced viral infectivity approximately two-fold. These findings have two important implications:

Firstly, they provide insight into the potential sequence of mutation acquisition. The simultaneous emergence of T478K and L452R mutations seems plausible. However, when considering the P681R mutation [61, 62], our data suggest that L452R might have been acquired prior to T478K. This inference is based on the observation that in the presence of P681R, the T478K mutation appears to impair viral infectivity, possibly due to reduced viral fitness (Fig. 2C). Secondly, our results offer a potential explanation for the Delta variant's ability to replace the Kappa variant and achieve viral dominance. The presence of both L452R and T478K mutations confers enhanced infectivity to the Delta variant (Fig. 2C), potentially providing a competitive advantage over the Kappa variant. These observations contribute to our understanding of the evolutionary dynamics driving SARS-CoV-2 variant succession and highlight the complex interplay between multiple mutations in determining viral fitness and transmissibility.

Previous studies have reported that the P681R mutation enhances viral infectivity by promoting spike protein cleavage mediated by cellular proteases [61, 62], thereby facilitating direct fusion between the viral envelope and cell membrane. However, our findings diverge from these reports, as we did not observe a significant role for the P681R mutation in enhancing viral infectivity or immune evasion within our experimental system. This discrepancy may be attributed to several factors, including the inherent characteristics of the pseudovirus system employed, the specific cell types utilized in our assays, and other experimental variables. It is important to note that the pseudovirus system, while valuable for studying specific aspects of viral biology, may not fully recapitulate all features of live virus infection. Given these conflicting observations, further comprehensive analysis is warranted to elucidate the precise role of the P681R mutation in SARS-CoV-2 biology. Such investigations should aim to reconcile the differences observed between various experimental systems and provide a more nuanced understanding of how this mutation contributes to viral fitness and pathogenesis in diverse cellular contexts.

In summary, our findings demonstrate that the mutations present in the spike proteins of the Delta and Kappa variants confer distinct advantages in terms of viral infectivity or antibody evasion. In the absence of widespread antiviral immunity, whether induced by vaccination or natural infection, enhanced infectivity may provide a relative advantage over antibody evasion. However, in the context of mass vaccination programs that potentially enable herd immunity, the evolutionary dynamics and competitive outcomes could differ significantly. These observations underscore the importance of increasing vaccination coverage to minimize the risk of variant emergence. By reducing the pool of susceptible individuals and limiting viral transmission, widespread vaccination can potentially constrain the opportunities for the virus to acquire and propagate advantageous mutations.

Our study highlights the complex interplay between viral evolution, host immunity, and public health interventions in shaping the trajectory of the SARS-CoV-2 pandemic. It emphasizes the critical need for continued surveillance, genomic monitoring, and adaptive vaccination strategies to mitigate the impact of emerging variants and guide effective public health responses.

## Figures and Tables

**Table 1:** Mutations in the spike protein of B.1.617 sub-lineage.

List of mutations		
Lineage	Mutations	
	in RBD	Other region than RBD
B.1.617.1 (Kappa variant)	L452R, E484Q	T91I, G142D, E154K, D614G, P681R, Q1071H
B.1.617.2 (Delta variant)	L452R, T478K	T19R, Δ157-158, D614G, P681R, D950N

The key mutations in the spike protein of B.1.617.1 and B.1.617.2 variants are underlined: the mutations in the RBD (receptor binding domain, L452R and T478K) and the S1/S2 cleavage site (the P681R mutation is primarily found in Delta variants only) as well as the D614G, which are found in almost all variants since the Alpha variant.

## References

[1] Lamers MM, Haagmans BL (2022). SARS-CoV-2 pathogenesis. Nat. Rev. Microbiol..

[2] Hu B, Guo H, Zhou P, Shi Z-L (2021). Characteristics of SARS-CoV-2 and COVID-19. Nat. Rev. Microbiol..

[3] Ogando NS, Zevenhoven-Dobbe JC, van Der Meer Y, Bredenbeek PJ, Posthuma CC, Snijder EJ (2020). The enzymatic activity of the nsp14 exoribonuclease is critical for replication of MERS-CoV and SARS-CoV-2. J. Virol..

[4] Tahir M (2021). Coronavirus genomic nsp14‐ExoN, structure, role, mechanism, and potential application as a drug target. J. Med. Virol..

[5] Takada K, Ueda MT, Shichinohe S, Kida Y, Ono C, Matsuura Y (2023). Genomic diversity of SARS-CoV-2 can be accelerated by mutations in the nsp14 gene. iScience.

[6] Mahdy MA, Younis W, Ewaida Z (2020). An overview of SARS-CoV-2 and animal infection. Front. Vet. Sci..

[7] Meekins DA, Gaudreault NN, Richt JA (2021). Natural and experimental SARS-CoV-2 infection in domestic and wild animals. Viruses.

[8] Chakraborty C, Bhattacharya M, Islam MA, Zayed H, Ohimain EI, Lee SS (2024). Reverse zoonotic transmission of SARS-CoV-2 and monkeypox virus: a comprehensive review. J. Microbiol..

[9] Machkovech HM, Hahn AM, Wang JG, Grubaugh ND, Halfmann PJ, Johnson MC (2024). Persistent SARS-CoV-2 infection: significance and implications. Lancet Infect. Dis..

[10] Wang X, Lu L, Jiang S (2024). SARS-CoV-2 evolution from the BA. 2.86 to JN. 1 variants: unexpected consequences. Trends Immunol..

[11] Mlcochova P, Kemp SA, Dhar MS, Papa G, Meng B, Ferreira IA (2021). SARS-CoV-2 B.1.617. 2 Delta variant replication and immune evasion. Nature.

[12] Li B, Deng A, Li K, Hu Y, Li Z, Shi Y (2022). Viral infection and transmission in a large, well-traced outbreak caused by the SARSCoV-2 Delta variant. Nat. Commun..

[13] Sigal A, Milo R, Jassat W (2022). Estimating disease severity of Omicron and delta SARS-CoV-2 infections. Nat. Rev. Immunol..

[14] Fisman DN, Tuite AR (2021). Evaluation of the relative virulence of novel SARS-CoV-2 variants: a retrospective cohort study in Ontario, Canada. CMAJ.

[15] Thomson EC, Rosen LE, Shepherd JG, Spreafico R, da Silva Filipe A, Wojcechowskyj JA (2021). Circulating SARS-CoV-2 spike N439K variants maintain fitness while evading antibody-mediated immunity. Cell.

[16] Hsieh CL, Goldsmith JA, Schaub JM, DiVenere AM, Kuo HC, Javanmardi K (2020). Structure-based design of prefusionstabilized SARS-CoV-2 spikes. Science.

[17] Mlcochova P, Kemp SA, Dhar MS, Papa G, Meng B, Ferreira I (2021). SARS-CoV-2 B.1.617.2 Delta variant replication and immune evasion. Nature.

[18] Davis C, Logan N, Tyson G, Orton R, Harvey WT, Perkins JS (2021). Reduced neutralisation of the Delta (B.1.617.2) SARS-CoV-2 variant of concern following vaccination. PLoS Pathog..

[19] Garcia-Beltran WF, Lam EC, St Denis K, Nitido AD, Garcia ZH, Hauser BM (2021). Multiple SARS-CoV-2 variants escape neutralization by vaccine-induced humoral immunity. Cell.

[20] Newman J, Thakur N, Peacock TP, Bialy D, Elrefaey AME, Bogaardt C (2022). Neutralizing antibody activity against 21 SARSCoV-2 variants in older adults vaccinated with BNT162b2. Nat. Microbiol..

[21] Lauring AS, Tenforde MW, Chappell JD, Gaglani M, Ginde AA, McNeal T (2022). Clinical severity of, and effectiveness of mRNA vaccines against, covid-19 from omicron, delta, and alpha SARS-CoV-2 variants in the United States: prospective observational study. BMJ.

[22] Bian L, Gao Q, Gao F, Wang Q, He Q, Wu X (2021). Impact of the Delta variant on vaccine efficacy and response strategies. Expert Rev. Vaccines.

[23] Lee HD, Chun J, Kim S, Aleksandra N, Lee C, Yoon D (2024). Comparative biodistribution study of baculoviral and adenoviral vector vaccines against SARS-CoV-2. J. Microbiol. Biotechnol..

[24] Myoung J (2022). Two years of COVID-19 pandemic: where are we now?. J. Microbiol..

[25] Mattoo SU, Myoung J (2022). T cell responses to SARS-CoV-2 in humans and animals. J. Microbiol..

[26] McCallum M, Walls AC, Sprouse KR, Bowen JE, Rosen LE, Dang HV (2021). Molecular basis of immune evasion by the Delta and Kappa SARS-CoV-2 variants. Science.

[27] Ren W, Ju X, Gong M, Lan J, Yu Y, Long Q (2022). Characterization of SARS-CoV-2 variants B.1.617. 1 (Kappa), B. 1.617. 2 (Delta), and B. 1.618 by cell entry and immune evasion. mBio.

[28] Jeong JY, Yim HS, Ryu JY, Lee HS, Lee JH, Seen DS (2012). One-step sequence- and ligation-independent cloning as a rapid and versatile cloning method for functional genomics studies. Appl. Environ. Microbiol..

[29] Islam MN, Lee KW, Yim HS, Lee SH, Jung HC, Lee JH (2017). Optimizing T4 DNA polymerase conditions enhances the efficiency of one-step sequence- and ligation-independent cloning. Biotechniques.

[30] Bae S, Lee JY, Myoung J (2020). Chikungunya virus nsP2 impairs MDA5/RIG-I-mediated induction of NF-kappaB promoter activation: A potential target for virus-specific therapeutics. J. Microbiol. Biotechnol..

[31] Lee JY, Nguyen TTN, Myoung J (2020). Zika virus-encoded NS2A and NS4A strongly downregulate NF-kappaB promoter activity. J. Microbiol. Biotechnol..

[32] Lee HJ, Park M, Choi H, Nowakowska A, Moon C, Kwak JH (2021). Pine needle extract applicable to topical treatment for the prevention of human papillomavirus infection. J. Microbiol. Biotechnol..

[33] Zhou YF, Nie JJ, Shi C, Ning K, Cao YF, Xie Y (2022). Expression and immunogenicity of SARS-CoV-2 virus-like particles based on recombinant truncated HEV-3 ORF2 capsid protein. J. Microbiol. Biotechnol..

[34] Kori M, Kasavi C, Arga KY (2024). Exploring COVID-19 pandemic disparities with transcriptomic meta-analysis from the perspective of personalized medicine. J. Microbiol..

[35] Kim TH, Bae S, Goo S, Myoung J (2023). Distinctive combinations of RBD mutations contribute to antibody evasion in the case of the SARS-CoV-2 beta variant. J. Microbiol. Biotechnol..

[36] Shim W, Lee A, Lee JH (2024). The role of extracellular vesicles in pandemic viral infections. J. Microbiol..

[37] Lee HJ, Choi H, Nowakowska A, Kang LW, Kim M, Kim YB (2023). Antiviral activity against SARS-CoV-2 variants using in silico and in vitro approaches. J. Microbiol..

[38] Kim S (2022). COVID-19 drug development. J. Microbiol. Biotechnol..

[39] Lee SJ, Kim YJ, Ahn DG (2022). Distinct molecular mechanisms characterizing pathogenesis of SARS-CoV-2. J. Microbiol. Biotechnol..

[40] Moon JS, Lee W, Cho YH, Kim Y, Kim GW (2024). The Significance of N6-methyladenosine RNA methylation in regulating the hepatitis B virus life cycle. J. Microbiol. Biotechnol..

[41] Kim WJ, Lee AR, Hong SY, Kim SH, Kim JD, Kim SJ (2024). Characterization of a small plaque variant derived from genotype V Japanese encephalitis virus clinical isolate K15P38. J. Microbiol. Biotechnol..

[42] Huynh DT, Chathuranga WAG, Chathuranga K, Lee JS, Kim CJ (2024). Mucosal administration of *Lactobacillus casei* surfacedisplayed HA1 induces protective immune responses against avian influenza A virus in mice. J. Microbiol. Biotechnol..

[43] Cho SY, Lee YJ, Jung SM, Son YM, Shin CG, Kim ET (2024). Establishment of a dual-vector system for gene delivery utilizing prototype foamy virus. J. Microbiol. Biotechnol..

[44] Saville JW, Mannar D, Zhu X, Srivastava SS, Berezuk AM, Demers JP (2022). Structural and biochemical rationale for enhanced spike protein fitness in delta and kappa SARS-CoV-2 variants. Nat. Commun..

[45] Korber B, Fischer WM, Gnanakaran S, Yoon H, Theiler J, Abfalterer W (2020). Tracking changes in SARS-CoV-2 spike: evidence that D614G increases infectivity of the COVID-19 virus. Cell.

[46] Ozono S, Zhang Y, Ode H, Sano K, Tan TS, Imai K (2021). SARS-CoV-2 D614G spike mutation increases entry efficiency with enhanced ACE2-binding affinity. Nat. Commun..

[47] Zhang J, Cai Y, Xiao T, Lu J, Peng H, Sterling SM (2021). Structural impact on SARS-CoV-2 spike protein by D614G substitution. Science.

[48] Harvey WT, Carabelli AM, Jackson B, Gupta RK, Thomson EC, Harrison EM (2021). SARS-CoV-2 variants, spike mutations and immune escape. Nat. Rev. Microbiol..

[49] Mader A-L, Tydykov L, Glück V, Bertok M, Weidlich T, Gottwald C (2022). Omicron's binding to sotrovimab, casirivimab, imdevimab, CR3022, and sera from previously infected or vaccinated individuals. IScience.

[50] Nie J, Li Q, Wu J, Zhao C, Hao H, Liu H (2020). Establishment and validation of a pseudovirus neutralization assay for SARSCoV-2. Emerg. Microbes Infect..

[51] Neerukonda SN, Vassell R, Herrup R, Liu S, Wang T, Takeda K (2021). Establishment of a well-characterized SARS-CoV-2 lentiviral pseudovirus neutralization assay using 293T cells with stable expression of ACE2 and TMPRSS2. PLoS One.

[52] Zhang J, Xiao T, Cai Y, Lavine CL, Peng H, Zhu H (2021). Membrane fusion and immune evasion by the spike protein of SARSCoV-2 Delta variant. Science.

[53] Wang Y, Liu C, Zhang C, Wang Y, Hong Q, Xu S (2022). Structural basis for SARS-CoV-2 Delta variant recognition of ACE2 receptor and broadly neutralizing antibodies. Nat. Commun..

[54] Hou YJ, Chiba S, Halfmann P, Ehre C, Kuroda M, Dinnon III KH (2020). SARS-CoV-2 D614G variant exhibits efficient replication ex vivo and transmission in vivo. Science.

[55] Zhou B, Thao TTN, Hoffmann D, Taddeo A, Ebert N, Labroussaa F (2021). SARS-CoV-2 spike D614G change enhances replication and transmission. Nature.

[56] Verghese M, Jiang B, Iwai N, Mar M, Sahoo MK, Yamamoto F (2021). A SARS-CoV-2 variant with L452R and E484Q neutralization resistance mutations. J. Clin. Microbiol..

[57] Ferreira IA, Kemp SA, Datir R, Saito A, Meng B, Rakshit P (2021). SARS-CoV-2 B.1.617 mutations L452R and E484Q are not synergistic for antibody evasion. J. Infect. Dis..

[58] Motozono C, Toyoda M, Zahradnik J, Saito A, Nasser H, Tan TS (2021). SARS-CoV-2 spike L452R variant evades cellular immunity and increases infectivity. Cell Host Microbe.

[59] Deng X, Garcia-Knight MA, Khalid MM, Servellita V, Wang C, Morris MK (2021). Transmission, infectivity, and antibody neutralization of an emerging SARS-CoV-2 variant in California carrying a L452R spike protein mutation. medRxiv [Preprint].

[60] Kannan SR, Spratt AN, Cohen AR, Naqvi SH, Chand HS, Quinn TP (2021). Evolutionary analysis of the Delta and Delta Plus variants of the SARS-CoV-2 viruses. J. Autoimmun..

[61] Saito A, Irie T, Suzuki R, Maemura T, Nasser H, Uriu K (2022). Enhanced fusogenicity and pathogenicity of SARS-CoV-2 Delta P681R mutation. Nature.

[62] Liu Y, Liu J, Johnson BA, Xia H, Ku Z, Schindewolf C (2022). Delta spike P681R mutation enhances SARS-CoV-2 fitness over Alpha variant. Cell Rep..

